# Extracorporeal irradiated tumor bone: A reconstruction option in diaphyseal Ewing’s sarcomas

**DOI:** 10.4103/0019-5413.69310

**Published:** 2010

**Authors:** Ajay Puri, Ashish Gulia, MG Agarwal, NA Jambhekar, S Laskar

**Affiliations:** Department of Orthopedic Oncology, Tata Memorial Hospital, Mumbai, India; 1 Department of Pathology, Tata Memorial Hospital, Mumbai, India; 2 Department of Radiation Oncology, Tata Memorial Hospital, Mumbai, India

**Keywords:** Ewing’s sarcomas, extracorporeal irradiation, limb salvage

## Abstract

**Background:**

Limb salvage in extremity tumors is now established as an oncologically safe option without compromising long-term survival. En bloc resection followed by extracorporeal radiation and reimplantation is a biological reconstruction option in diaphyseal Ewing’s sarcomas. We analyzed the results of 12 cases of diaphyseal Ewing’s sarcomas treated using this modality.

**Materials and Methods:**

Between March 2006 and March 2008, 12 patients with Ewing’s sarcoma underwent enbloc resection and reconstruction, with reimplantation of the sterilized tumor bone, after extracorporeal irradiation. There were eight males and four females, with a mean age of 14 years (range 2 to 22 years). The femur was the most common bone involved (n=8) followed by the tibia and the humerus (two cases each). All these patients were non-metastatic at presentation and received chemotherapy as per the existing hospital protocol. The mean length of the bone resected was 20 cm (range 11 to 25 cm). The specimen was irradiated with 50 Gy prior to reimplantation and stabilized with the host bone, using suitable internal fixation. Standard biplanar radiographs were assessed for evidence of union on the follow-up visits. The functional status was assessed using the Musculoskeletal Tumor Society Scoring system at the time of the last follow up. The mean follow up duration was 29 months (range 12 to 57 months).

**Results:**

Two patients (17%) had early infection with graft removal, hence are excluded from any analysis of union, however they are included when analysing complications such as infection. Rest 10 cases were analyzed for bony union at the osteotomy sites. Sixteen (84%) of the 19 osteotomy sites united primarily, without any intervention. Implant failure and non-union was seen at three diaphyseal osteotomy sites. The average time for union of all osteotomy sites was 7.2 months (range 3 to 13 months).The average time for union of the metaphyseal osteotomy sites was 5.9 months (range 3 to 12 months) and of diaphyseal osteotomy sites was 8.3 months (range 4 to 13 months). The mean Musculoskeletal Tumor Society Score was 27 (range 19 to 30) with a mean of 27. Nine of the ten patients with lower limb involvement were independent ambulators without additional aids. At the time of the last review, six patients were free of disease and six patients had died from the disease. There were two recurrences around the operative site. Both were associated with disseminated disease and in both the recurrences were in the soft tissue, away from the irradiated graft.

**Conclusion:**

Extracorporeal irradiation is a useful, convenient technique for limb salvage in diaphyseal Ewing’s sarcomas when there is reasonable residual bone stock. It is oncologically safe and has good functional results. A radiation dose of 50 Gy for sterilizing the bone ensures adequate tumor kill, while minimizing the deleterious effects on the biomechanical and biological properties of the bone. The use of appropriate implants for adequate internal fixation and supplementary bone grafting at the index surgery may help reduce the need for subsequent additional interventions to achieve union. The limitations of this procedure are that it is not applicable in tumor bones that are structurally weak and in bones with pathological fractures.

## INTRODUCTION

In an earlier era local treatment of non-metastatic Ewing’s sarcomas with surgery (generally amputation) or irradiation alone was ineffective, as the large majority of patients died within five years, with disseminated disease.^1^,^2^ With the advent of effective chemotherapy the outcome of localized Ewing’s sarcomas improved significantly. The advantages of surgery, compared to radiotherapy alone, for local control, have also been adequately demonstrated in numerous studies, advocating a multidisciplinary approach to these lesions.^3^–^5^

Limb salvage in extremity tumors is now established as an oncologically safe option, without compromising long-term survival. Most bone tumors occur in the metaphyseal area of the bones and resection with a wide margin generally involves sacrifice of the adjoining articular surface. Megaprosthesis provides an effective reconstruction option in a majority of these cases, with good functional results.^6^,^7^ Tumors in the diaphysis are relatively uncommon and in most of these it may be possible to achieve adequate margins without sacrificing the adjacent articular surfaces. Although there are reports of diaphyseal prosthesis, this intercalary gap can be reconstructed using other methods too. Reconstruction using biological options like autografts, allografts, and bone transport have shown good functional results.^8^,^9^

In recent times, there has been a lot of interest in using the patient’s own tumor bone and reimplanting it after it has been sterilized. The described methods of sterilization have included the use of autoclaving, microwave, pasteurizing, liquid nitrogen, and radiotherapy (extracorporeal radiotherapy).^10^–^14^ The principle is the same; the tumor-bearing bone is excised enbloc, all soft tissues and macroscopic tumor removed, and the remaining bone sterilized by any of the above mentioned methods before being reimplanted. We have been using extracorporeal radiation and reimplantation for bony tumors since 2006, and have done this procedure in more than 45 patients. In this article we analyze and present the results of 12 cases of diaphyseal Ewing’s sarcomas treated using this modality. We observed the complications arising after reconstruction, time taken for union, and the functional outcomes after reconstruction.

## MATERIALS AND METHODS

Between March 2006 and March 2008, 12 patients with Ewing’s sarcoma underwent enbloc resection and reconstruction with reimplantation of sterilized tumor bone after extracorporeal irradiation [Table 1]. These patients were identified by a retrospective review of a prospectively maintained database. The medical records, imaging, disease status, and functional status were reviewed. There were eight males and four females with a mean age of 14 years (range 2 to 22 years). The femur was the most common bone involved (n=8) followed by tibia and humerus (two cases each).

**Table 1:** Clinical details of patients

Case	Age	Sex	Bone	RL (cms)	Diaphyseal union (months)	Metaphyseal union (months)	Complications	Follow-up (months)	MSTS score	Current status
1	9	M	Tibia	17	7	7	None	15	28	Dead pulmonary metastasis
2	19	M	Femur	18	12	12	None	39	29	Local recurrence (36 months) + dead disseminated disease
3	22	F	Femur	19	4	6	None	18	27	Dead disseminated disease
4	16	F	Femur	22	–	–	Infection	57	28	Alive — Independent ambulation on nail cement spacer
5	16	M	Femur	25	–	–	Infection	44	19	Alive — Flail limb
6	10	M	Humerus	12	10	5	Radial N Palsy, implant failure (6 months), non Union	12	24	Dead pulmonary metastasis
7	2	M	Femur	11	6	3	None	36	30	Alive
8	19	M	Humerus	21	13	6	Implant failure (10 months), non-union	36	28	Alive
9	14	F	Femur	30	Bipolar prosthesis	3	None	18	24	Local recurrence (13 months) + dead disseminated disease
10	11	M	Tibia	23	6	6	Plate exposed — Flap cover	34	30	Alive
11	15	F	Femur	25	6	6	None	18	28	Dead — Refused post op chemotherapy pulmonary metastasis
12	16	M	Femur	24	11	6	Implant failure (7 months), non-union	22	26	Alive

MSTS: Musculoskeletal Tumor Society

The average duration of the symptoms was 3.5 months (range 2 to 12 months). A tissue diagnosis was obtained preoperatively from all the patients. In case slides and blocks (n = 6) from prior intervention were available, these were reviewed at our institute. If not, a biopsy (n = 6) was performed. In a large majority of the cases we preferred a core needle biopsy to obtain the tissue. The biopsy results were discussed in a multidisciplinary meeting, which included a radiologist and a pathologist specializing in bone tumors. Prior to surgery all the 12 patients underwent a thorough oncological assessment to determine the extent of local disease and the presence of distant metastases. Staging studies, including plain radiographs and MRI of the limb, CT scans of the chest, and total body scintigraphy were performed. All these patients were non-metastatic at presentation. All the patients received neoadjuvant (induction) and adjuvant (maintenance) chemotherapy as per the existing hospital protocol.

The MRI was used to define the extent of the lesion, the involvement of the soft tissues, relation to the neurovascular bundle, and the level of transection of the bone. The primary goal of surgery was complete excision of the tumor, with preservation of the limb. A 2 – 3 cm marrow margin as calculated on the T1 WI MRI image was considered as an adequate resection margin. The mean length of the bone resected was 20 cm (range 11 to 25 cms).

After tumor excision, a sample of the marrow was sent for a frozen section from both residual ends of the host bone, to confirm clear margins. The resected specimen was then transferred to a separate sterile trolley, away from the main operative field to avoid any contamination of the operative field. Under aseptic precautions all the soft tissue including the periosteum was stripped from the bone after inking the closest soft tissue margin. This inking of margins helped the pathologist report on the adequacy of resection in the final histopathology report, which otherwise would not have been possible.

**Figure 1:**
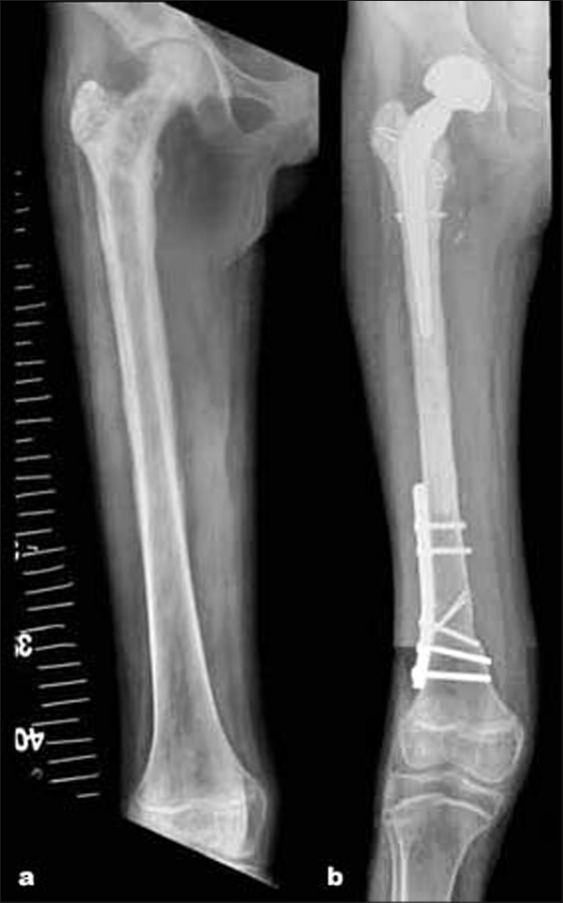
(a) Anteroposterior radiograph showing proximal and diaphyseal femur Ewing’s sarcoma (case 9). (b) Extracorporeal irradiation and reimplantation, with cemented bipolar prosthesis inserted at the proximal end. Nine months follow up showing prosthesis *in situ* and distal osteotomy united

The bone specimen was lavaged with normal saline and wrapped in Vancomycin-soaked mops. This was then wrapped in sterile polyethylene surgical drapes in two separate layers and placed in a sterile container, which was sent for extracorporeal irradiation. The resected bone segment enclosed in the sterile pack was irradiated to a dose of 50 Gy / 1 fraction, prescribed to the midplane of the specimen, using 6 MV photons or ^60^Cobalt γ rays with parallel opposing portals. The mean treatment time for delivery of 50 Gy was 28 minutes (range 24 to 36 minutes). The average time for transfer of the graft from the operating room to completion of the radiation therapy procedure and return of the irradiated specimen to the operating room was 55 minutes (range 45 to 75 minutes). After returning to the operative room the excised bone was prepared for reimplantation. The marrow contents were removed by reaming and the bone specimen was lavaged with a high speed pulsatile lavage system to remove the residual marrow tissue. Bone cement was packed in the medullary cavity of the radiated graft. Care was taken to ensure that the cement was just short of the osteotomy sites, so as not to interfere with the bony apposition and eventual union. The specimen was realigned with the host bone and stabilized with suitable extramedullary internal fixation. This included standard dynamic compression plates (n=3), reconstruction plates (n=2), custom-made plates (n=5), and a locking compression plate (n=1). In one case involving the proximal femur, a bipolar prosthesis was inserted at one end to articulate with the acetabulum and the distal osteotomy was stabilized with a locking compression plate [Figure 1].

The average surgical time for the procedure was seven hours (range 6 to 8.5 hours). The average blood loss was 1050 ml (range 300 ml to 2300 ml). None of the patients received postoperative radiotherapy. The patients were allowed to mobilize after surgery with the use of an appropriate brace or cast. Postoperative range of motion of the adjacent joint was permitted according to the stability of the entire construct. In cases of lower limb disease, guarded partial weight bearing using walking assists, progressed to eventual full weight bearing, once evidence of the bony union was seen on the radiographs. Standard biplanar radiographs were assessed. Bridging across three of four cortices in biplanar radiographs was considered evidence of the union.

The patients were asked to follow up every three months for the first two years and every six months, subsequently. Besides screening for disease surveillance, biplanar radiographs of the local part were assessed at each visit. The functional status was assessed using the Musculoskeletal Tumor Society Scoring system at the time of the last follow-up.^15^ The mean follow-up for all patients was 29 months (range 12 to 57 months). The mean follow-up duration for survivors was 38 months (range 22 to 57 months). The mean follow-up duration for non survivors was 20 months (range 12 to 39 months).

## RESULTS

All resection margins were free of tumor on histopathology, as evaluated on the intraoperative frozen specimens and the final definitive histopathology. One patient had transient radial nerve palsy and eventually recovered, and one patient (case 10) had postoperative skin necrosis requiring a flap cover. Two patients (17%) had infection (case 4,5). In one of them the graft was removed immediately and non-biological reconstruction was done, using a nail cement spacer. The infection subsided and the patient did not require any additional procedures. In the other case repeated attempts to salvage the graft with lavage were unsuccessful. The graft was eventually removed and the defect reconstructed using a nail cement spacer. The infection persisted, necessitating removal of the nail cement spacer too. Currently the patient has a flail limb and ambulates on crutches. In both cases the organisms were MRSA resistant to vancomycin. The infection rate was similar to that with the use of bank allografts, which was the other modality of reconstruction we used for diaphyseal defects.

Implant failure and non-union was seen in three cases at three diaphyseal osteotomy sites (Case no: 6,8,12). All required open reduction and repeat internal fixation, with thicker plates and bone grafting, which eventually united at 10, 11, and 13 months after index surgery. Thus additional procedures were required in six of 12 patients (50%). These included open reduction and internal fixation with bone grafting in three patients, wound lavage and graft removal in two patients, and flap cover for skin necrosis in one patient.

After excluding two cases of infection where the graft was removed, 10 cases were analyzed for bony union at the osteotomy sites. In one of these a bipolar prosthesis was inserted at one end. Thus 19 osteotomy sites were analyzed for bony union in 10 patients. There were 10 diaphyseal and nine metaphyseal sites. Sixteen (84%) of 19 osteotomy sites united primarily without any intervention. The average time for union of all osteotomy sites was 7.2 months (range 3 to 13 months). The average time for union of metaphyseal osteotomy sites was 5.9 months (range 3 to 12 months) and of the diaphyseal osteotomy sites was 8.3 months (range 4 to 13 months).

The functional status was determined at the final follow-up using the Musculoskeletal Tumor Society scoring system. This was based on the analysis of three factors (pain, functional activities, and emotional acceptance) pertinent to the patient as a whole and three factors specific to either the upper limb or lower limb. For the upper limb, positioning of the hand, manual dexterity, and lifting ability were assessed, while for the lower limb use of supports for ambulation, walking ability, and gait were assessed. For each of the six factors, values of 0 to 5 were assigned based on the established criteria. The result was expressed as a sum total, with a maximum score of 30, and as a percentage of the expected normal function for the patient. The Musculoskeletal Tumor Society Score for patients evaluated at their last follow-up ranged from 19 to 30 months with a mean of 27 months. Nine of the 10 patients with lower limb involvement were independent ambulators, without additional aids.

At the time of the last review, six patients were free of the disease and six patients had died from the disease. Patients with disseminated disease at time of last follow up were referred for best supportive care and final status of these patients was confirmed by telephonic contact. Mean follow up of non survivors was 20 months. Of those who died, one patient refused post surgery maintenance chemotherapy and succumbed to pulmonary metastasis. Her osteotomy united at 7 months. Two others developed pulmonary metastasis and three patients developed disseminated disease (distant failure at multiple sites). There were two recurrences around the operative site. Both were associated with disseminated disease and in both, the recurrences were in the soft tissue, away from the irradiated graft. No recurrence occurred in the reimplanted bone.

## DISCUSSION

Reconstruction of long intercalary defects after tumor resection is a challenging proposition, especially in immunocompromised patients receiving cytotoxic chemotherapy. Custom-made diaphyseal implants provide the advantage of immediate weight bearing and ambulation, but are expensive and issues regarding loosening, wear, and breakage remain. Biological reconstructions provide a cost-efficient and more durable reconstruction option. The use of strut autografts and allografts has been well-documented in the reconstruction of these defects.^9^,^16^ The use of non-vascularized strut autografts is often limited by the length of the long resection gaps. Strut allografts, although a useful option, are limited by their availability, as very few surgeons in our country have access to bone bank facilities. Distraction osteogenesis, although described, requires the lengthy use of external pins and has thus not found universal application as the primary modality in patients undergoing treatment for malignant bone tumors.^17^

Reimplanting the sterilized tumor bone offers yet another option for reconstructing these defects. This procedure has a number of advantages, as it provides an anatomically size-matched graft for biological reconstruction. It is inexpensive and helps restore bone stock. The reimplanted bone acts as a scaffold for creeping substitution and incorporation. This procedure obviates the need for a bone bank and avoids the issues of allograft procurement and the risks associated with the use of allografts, such as, graft rejection and transmission of viral diseases. The limitations of this procedure are: It is not applicable in tumor bones, which are structurally weak, and in bones with pathological fractures. Various methods of sterilization have been described. Autoclaving the bone has the disadvantage of causing severe injury to bone proteins and the collagen matrix leading to considerable damage to the biological and biomechanical properties of the graft. Pasteurization has also been used successfully for sterilization of tumor bone, with good early results.^12^ Extracorporeal irradiation of autogenous tumor bone and its use for reimplantation was first described in 1968, by Spira and Lubin.^18^ Since then, there have been various authors who have advocated different radiotherapy doses for sterilizing the bone. We used a dose of 50 Gy, delivered in a single fraction, for sterilization of the tumor-bearing bone, as advocated by certain authors.^10^,^13^,^19^ They suggested that higher doses were not necessary for tumor sterilization. Higher doses of radiation would increase the total treatment time and also carry the additional risk of other possible detrimental effects such as reduction in strength, revascularization, and osteoconductive properties, thereby increasing the time for union and incorporation.^20^

**Figure 2:**
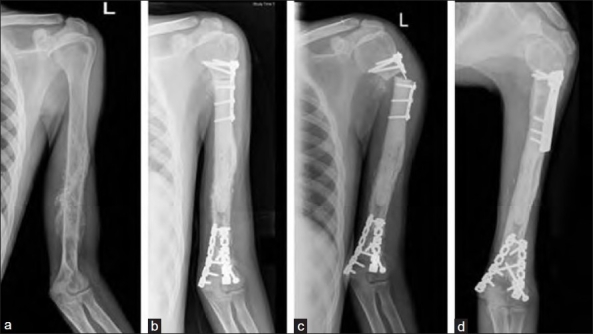
Anteroposterior radiograph of Humerus (a) showing Ewing’s sarcoma (case 8). (b) Postoperative radiograph after extracorporeal irradiation and reimplantation. (c) Non-union and implant failure at proximal diaphyseal osteotomy occured at 10 months followup. (d) Refixation with eventual union at 13 months follow-up radiograph at 30 months shows sound union

The rate of non-union in intercalary reconstructions with allografts has been reported to be as high as 63% and is higher in the diaphysis than in the metaphysis.^16^,^21^–^24^ In a series of extracorporeal irradiation autografting of the femur, non-union occurred in five of the 32 host-donor junctions (16%), and union occurred faster at the metaphyseal than at the diaphyseal junction.^25^ Our experience has been somewhat similar. We had non-union occur in three of the 19 host-donor junctions (16%). In our series the average time for union of the metaphyseal osteotomy sites was 5.9 months as against 8.3 months at the diaphyseal osteotomy sites. All three of our non-unions associated with implant failure were at the diaphyseal osteotomy sites [Figure 2].

These possibly occurred because of the use of improper implants.^26^ In two cases (cases 6 and 8) weak plates were used and in one case (case 12) a shorter than necessary locking compression plate was used, with an inadequate hold, in the host bone. All three eventually united after open reduction and repeat internal fixation with bone grafting. We did not primarily bone graft any of the osteotomy sites of the cases in this series.

Infection has been a major problem both in allografts and irradiated autografts.^27^ Infection rates for allografts vary from 6 to 17.6% and for irradiated autografts it ranges from 0 to 12%.^16^,^21^,^22^,^28^,^29^ We had infection occur in two of our 12 cases (17%). We were unable to salvage the graft in both, illustrating the disastrous consequences of infection in these large reconstructions. Wrapping the resected bone in Vancomycin soaked mops is an attempt to try and reduce the infection rate in these cases.

Local recurrence has been rarely reported after extracorporeal irradiation.^29^ We had two recurrences around the operative site. Both were associated with disseminated disease and in both, the recurrences were in the soft tissue, away from the irradiated graft. No recurrence occurred in the reimplanted bone. We also histopathologically analyzed both the grafts that were removed because of infection for any residual tumor. There was no evidence of disease in the retrieved specimens.

Variable fracture rates were reported in allografts.^16^,^30^–^32^ When fixation techniques resulted in cortical penetration of the allograft the fracture rate was 63%.^32^ The fracture rates for irradiated grafts were reported to be as high as 20%.^29^ This was in a series where a higher dose of radiation was used to sterilize the grafts. Using 50 Gy for radiation of the tumor-bearing bone and packing the medullary canal with bone cement had helped to avoid any early graft fractures in our series, although a longer follow-up would be required to validate the efficacy of this technique in reducing the incidence of graft fracture.^33^

The average functional score in our study, as evaluated by the Musculoskeletal Tumor Society, was 89%. This is encouraging when compared with other methods and with other series using an irradiated graft.^13^,^25^,^28^,^29^

One of the reported disadvantages of the use of extracorporeal irradiation is the lack of material available for the histopathological examination of the effects of chemotherapy and the adequacy of the resection margins.^25^ The marginal biopsies that we send intraoperatively, along with the inking of the closest soft tissue margin helps the pathologist report on the resection margins. The soft tissue component of the tumor retrieved prior to sending the bone for irradiation is adequate for the pathologist to comment on the presence or absence of the viable tumor. Thus both the effects of chemotherapy and the adequacy of the resection margins can be assessed in this technique. We do not use postoperative radiation (irrespective of necrosis) if the margins are clear. Therefore, if the marginal biopsies and inked margin in all specimens are reported negative for tumor, postoperative radiation is not considered, irrespective of whether the soft tissue component which is analyzed shows presence / absence of a viable tumor.

Reconstructing large resection gaps following tumor resection has always been challenging. Extracorporeal irradiation is a useful, convenient technique for limb salvage in diaphyseal Ewing’s sarcomas, when there is reasonable residual bone stock. Extracorporeal irradiation results in a relative increase in the total operating time, but is less expensive. It is oncologically safe and has good functional results. A radiation dose of 50 Gy for sterilizing the bone ensures adequate tumor kill, while minimizing deleterious effects on biomechanical and biological properties of the bone. The use of appropriate implants for adequate internal fixation and supplementary bone grafting at index surgery may help reduce the need for subsequent additional intervention to achieve a union. It is a highly technical procedure and the best result can be obtained in structured musculoskeletal oncology services.
